# Supplementary material for: Ecophysiological Responses of Tall Wheatgrass Germplasm to Drought and Salinity
Source: Plants (Basel). 2022 Jun 10;11(12):1548. doi: 10.3390/plants11121548 (PMC9227858; doi:10.3390/plants11121548)
Supplement: Supplementary file 1 [file plants-11-01548-s001.zip › Supplementary Materials FIGURES.pdf]

"Ecophysiological responses of tall wheatgrass germplasm to drought and salinity"

Supplementary Materials

Contains Figures: S1, S2, S3, S4, S5 and S6.

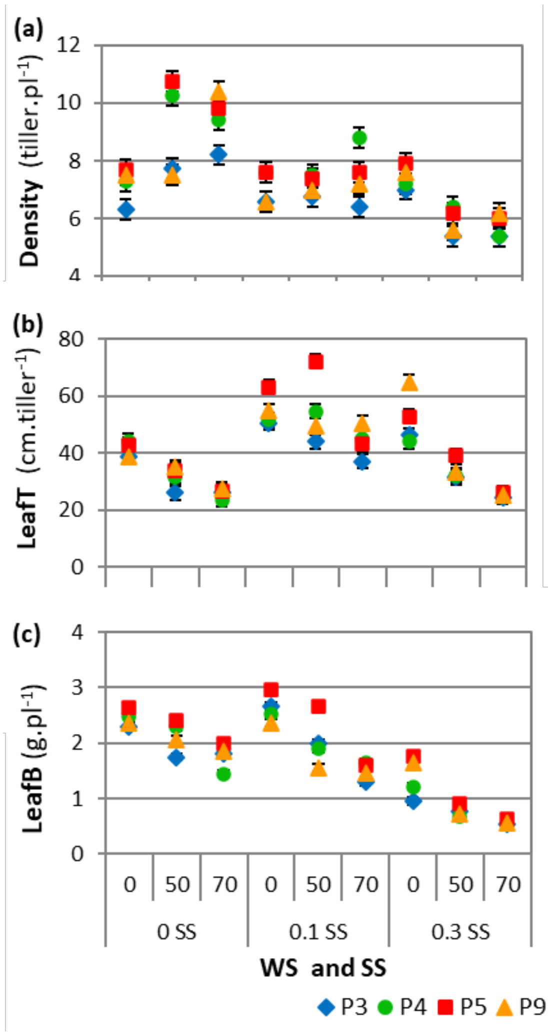

Figure S1. Density (a), green leaf length (LeafT, b) and leaf biomass (LeafB, c) of four accessions (P3, P4, P5, P9) grown under three drought levels (0WS, 50WS or 70WS correspond to 100%, 50%, 30% of water capacity, respectively) combined with three salinity levels (0SS, 0.1SS or 0.3SS correspond to 0.0, 0.1, 0.3M NaCl, respectively)

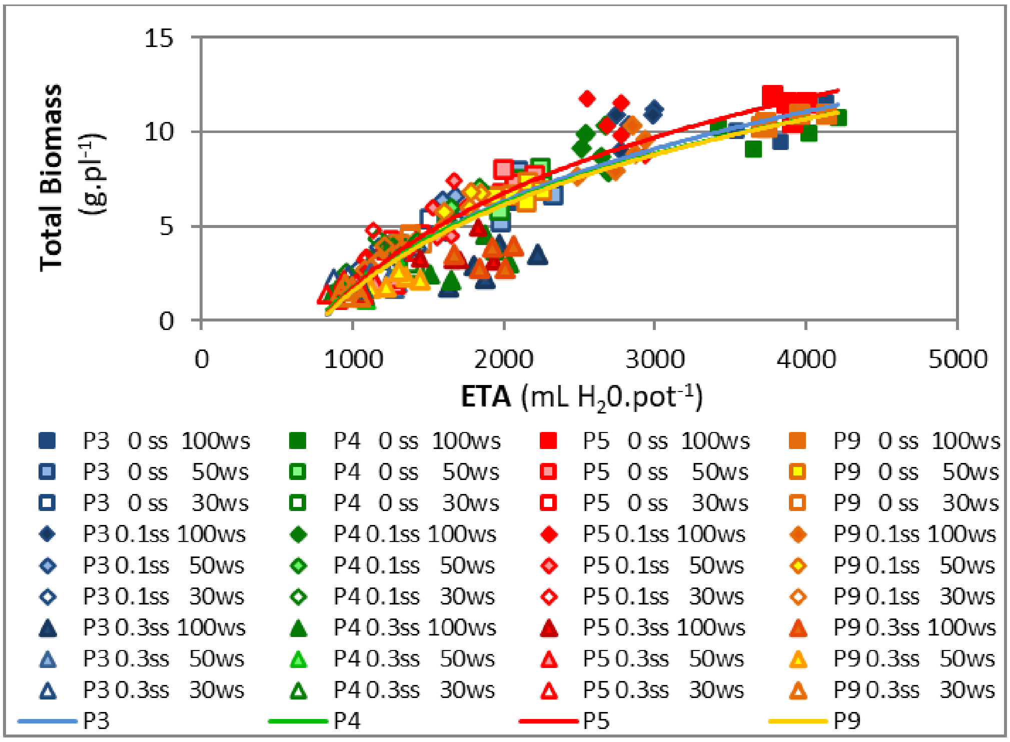

Figure S2. Relationship between total biomass and accumulated evapotranspiration (ETA) of four accessions (P3, P4, P5, P9) grown under three drought levels (0WS, 50WS or 70WS correspond to 100%, 50%, 30% of water capacity, respectively) combined with three salinity levels (0SS, 0.1SS or 0.3SS correspond to 0.0, 0.1, 0.3M NaCl, respectively). Color lines show the behavior of each accession, P3: y= 6.9ln(x) -45.8, $R^2 = 0.82$***, P4: y= 6.5ln(x) -42.9, $R^2 = 0.84$***, P5: y= 7.3ln(x) -48.6, $R^2 = 0.86$***, P9: y= 6.6ln(x) -44.0, $R^2 = 0.86$***, (*** $p < 0.001$).

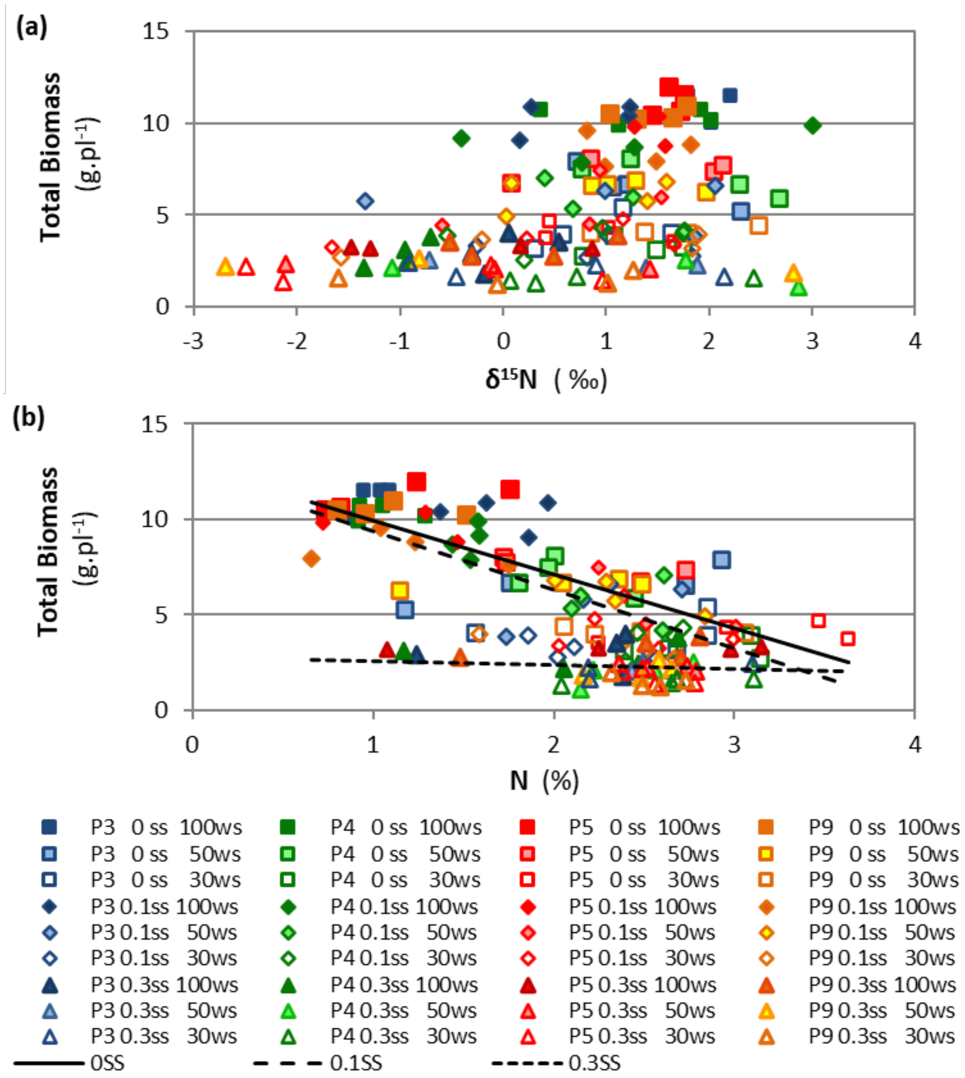

Figure S3. Relationship between total biomass and stable isotope of nitrogen ($\delta^{15}N$, a) or N concentration (b) of four accessions (P3, P4, P5, P9) grown under three drought levels (0WS, 50WS or 70WS correspond to 100%, 50%, 30% of water capacity, respectively) combined with three salinity levels (0SS, 0.1SS or 0.3SS correspond to 0.0, 0.1, 0.3M NaCl, respectively). Black lines of different sections show the behavior of three salinity levels for N%, 0SS: y=2.82x+12.7 R^2=0.62***, 1SS: y= -3.07x+12.45 R^2 =0.46*, 3SS: y=-0.20x+2.80 R^2 =0.02ns, (ns $p > 0.0500$, * $p < 0.0500$, *** $p < 0.0001$).

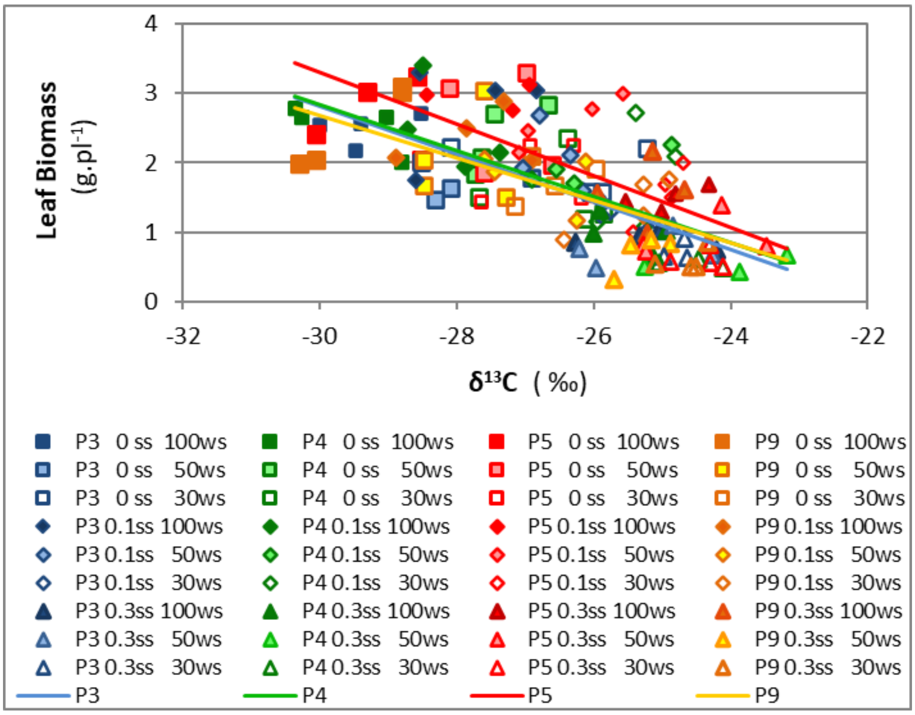

Figure S4. Relationship between leaf biomass and stable isotope of carbon ($\delta^{13}C$) of four accessions (P3, P4, P5, P9) grown under three drought levels (0WS, 50WS or 70WS correspond to 100%, 50%, 30% of water capacity, respectively) combined with three salinity levels (0SS, 0.1SS or 0.3SS correspond to 0.0, 0.1, 0.3M NaCl, respectively). Color lines show the behavior of each population, P3: $y = -0.34x - 7.54$, $R^2 = 0.50$*, P4: $y = -0.33x - 7.12$, $R^2 = 0.48$ns, P5: $y = -0.37x - 7.85$, $R^2 = 0.50$*, P9: $y = -0.30x - 6.44$, $R^2 = 0.43$ns, (ns $p > 0.0500$, * $p < 0.0500$).

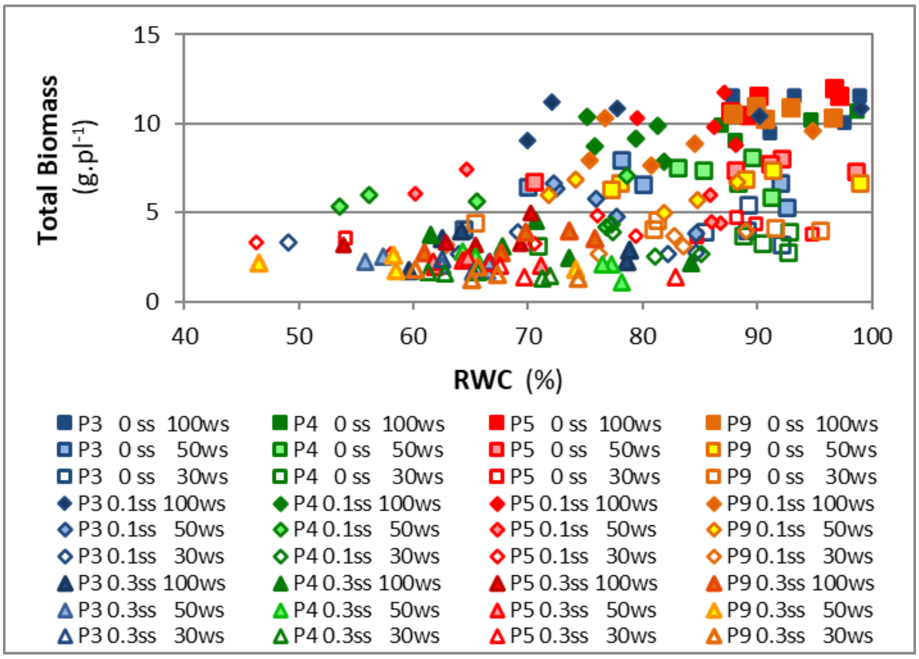

Figure S5. Relationship between total biomass and relative water content (RWC) of four accessions (P3, P4, P5, P9) grown under three drought levels (0WS, 50WS or 70WS correspond to 100%, 50%, 30% of water capacity, respectively) combined with three salinity levels (0SS, 0.1SS or 0.3SS correspond to 0.0, 0.1, 0.3M NaCl, respectively).

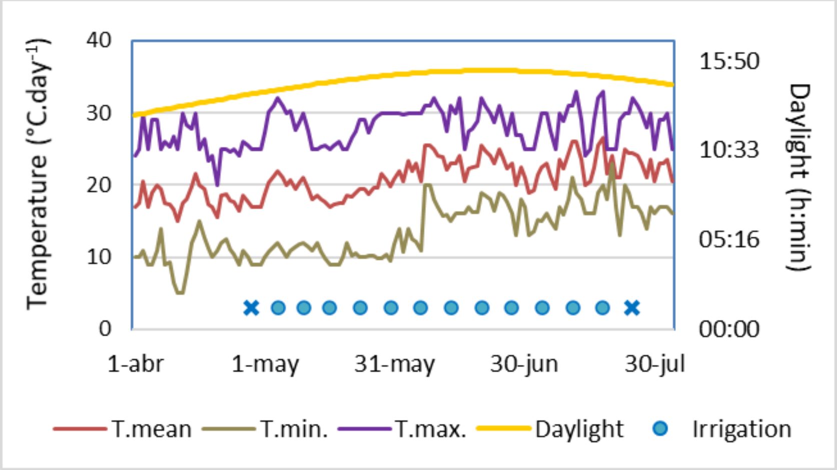

Figure S6. Mean, maximum and minimum daily temperatures recorded in the greenhouse and hours of daily light (daylight) during the experience. The beginning (April 28) and end (July 26) of the experiment (symbol X), and irrigation days (blue dots) were indicated.
