# Supplementary material for: Ecophysiological Responses of Tall Wheatgrass Germplasm to Drought and Salinity
Source: Plants (Basel). 2022 Jun 10;11(12):1548. doi: 10.3390/plants11121548 (PMC9227858; doi:10.3390/plants11121548)
Supplement: Supplementary file 1 [file plants-11-01548-s001.zip › Supplementary Materials TABLES.pdf]

"Ecophysiological responses of tall wheatgrass germplasm to drought and salinity"

Supplementary Materials

Contains Tables S1, S2, S3 and S4.

Table S1. Results of three-way factorial ANOVAs (four accessions, three salinity levels, three drought levels and their interactions) with five blocks for the morphoagronomic variables, p-values are considered significant when $p < 0.05$.

ANOVA	Density	LeafT	SLA	LeafB	StemB	DeadB	Dead%	TotalB	Height	Spike%	ETA	WUE	EC
Drought (WS)	0.3284	<0.0001	<0.0001	<0.0001	<0.0001	<0.0001	<0.0001	<0.0001	<0.0001	<0.0001	<0.0001	<0.0001	<0.0001
Salinity (SS)	<0.0001	<0.0001	<0.0001	<0.0001	<0.0001	<0.0001	<0.0001	<0.0001	<0.0001	<0.0001	<0.0001	<0.0001	<0.0001
Accession	0.0261	0.0244	0.8892	0.0010	0.0261	0.2076	0. 0482	0.0325	0.0456	0.0419	0.0074	0.0331	0.9539
WS × SS	0.0002	0.0093	0.2562	0.1327	<0.0001	0.0002	0.0232	<0.0001	<0.0001	<0.0001	<0.0001	<0.0001	<0.0001
WS × Accession	0.7031	0.4508	0.9004	0.2260	0.1044	0.1139	0.1656	0.1361	0.5352	0.4154	0.5246	0.4175	0.3280
SS × Accession	0.4645	0.5536	0.8263	0.3202	0.3557	0.1302	0.5248	0.8000	0.0662	0.0022	0.4525	0.6744	0.9194
WS × SS × Accession	0.7492	0.4801	0.3099	0.2362	0.1365	0.7990	0.3120	0.0900	0.5137	0.5251	0.7888	0.1401	0.9589

Density: tiller density; LeafT: length leaf per tiller; SLA: specific leaf area; LeafB, StemB, DeadB and TotalB: green-leaf, green-stem, dead and total biomass; Dead%: percentage of dead biomass; Heigth: plant height; Spike%: proportion of spiked tillers; ETA: accumulated evapotranspiration; WUE: water use efficiency; EC: electrical conductivity.

Table S2. Results of three-way factorial ANOVAs (four accession, three salinity levels, three drought levels and their interactions) with five blocks for physiological and isotopic variables, p-values are considered significant when $p < 0.05$.

ANOVA	RWC	Proline	K^+	Na^+	Cl^-	Na^+/K^+ ratio	N	$\delta^{13}C$	$\delta^{15}N$
Drought (WS)	<0.0001	<0.0001	<0.0001	0.1147	0.2091	<0.0001	<0.0001	<0.0001	0.5121
Salinity (SS)	<0.0001	<0.0001	<0.0001	<0.0001	<0.0001	<0.0001	<0.0001	<0.0001	<0.0001
Accessions	0.8082	0.5201	0.1454	0.7747	0.5658	0.0493	0.3991	0.0476	0.2823
WS × SS	0.3888	<0.0001	0.0110	0.5448	0.1905	0.0354	<0.0001	<0.0001	0.2848
WS × Accession	0.8060	0.7166	0.8249	0.7070	0.1183	0.4286	0.7237	0.4631	0.3451
SS × Accession	0.1066	0.6207	0.2750	0.6612	0.6047	0.2856	0.8440	0.9700	0.7090
WS × SS × Accession	0.5318	0.8353	0.6076	0.5087	0.4561	0.5114	0.7450	0.9805	0.7726

RWC: relative water content; $\delta^{13}C$: stable isotope of carbon; $\delta^{15}N$: nitrogen stable isotope.

Table S3. Results of three-way factorial ANCOVA (four accession, three salinity levels, three drought levels and their interactions) with covariates and repeated measures by time (t = 3) and four blocks for net photosynthetic rate (A), p-values are considered significant when $p < 0.05$.

ANOVA	A
t	<0.0001
WS	<0.0001
SS	<0.0001
Accession	0.6504
t × WS	0.0889
t × SS	<0.0001
t × Accession	0.9977
WS × SS	0.1986
WS × Pobla	0.7954
SS × Pobla	0.6466
t × WS × SS	0.0130
t × WS × Accession	0.9988
t × SS × Accession	0.9455
WS × SS × Accession	0.8493
t × WS × SS × Accession	0.9776
PAR	0.8230
T_air	0.5590
T_leaf	0.5557
CO_2	0.1798
Flow	0.3615
RH	0.0102

The covariates were photosynthetically active radiation (PAR), air and leaf temperature (Temp_A and Temp_L), CO_2 concentration, air flows (Flow) and relative humidity (RH).

Table S4: Broad-sense heritability (H^2 %) and standard errors (SE H^2) for morpho-agronomic, physiological and isotopic characters. Classification of heritability in low (< 40%), medium (40-59%) and high (60-79%) [45].

**Characters**	**Abbreviation**	**H^2**	**SE H^2**
Stable isotopes of ^{13}C	$\delta^{13}C$	76.9	10.0
Leaf biomass	LeafB	71.6	9.7
Tiller density	Density	70.8	11.8
Leaf length per tiller	LeafT	70.1	12.0
Na^+/K^+ ratio	Na/K	55.0	15.2
Plant height	Heigth	52.6	14.8
Total biomass	TotalB	50.9	15.2
Dead biomass	DeadB	50.3	15.3
K^+ concentration	K	49.6	15.9
Stem biomass	StemB	44.8	16.3
Free proline	Proline	41.7	16.4
Stable isotopes of ^{15}N	$\delta^{15}N$	37.4	16.5
Net photosynthetic rate at 85 days	A_t3	36.2	16.5
N concentration	N	34.3	16.5
Proportion of spiked tillers	Spike	30.8	16.3
Percentage of dead biomass	%Dead	26.8	16.0
Cl^- concentration	Cl	5.8	2.3
Specific leaf area	SLA	1.0	1.9
Relative water content	RWC	0.0	1.6
Na^+ concentration	Na	0.0	1.6
Net photosynthetic rate at 10 days	A_t1	0.0	1.6
Net photosynthetic rate at 45 days	A_t2	0.0	1.6
